# Longitudinal trajectories of peripheral blood lymphocyte subsets and PD-1 expression as dynamic prognostic markers in lung cancer

**DOI:** 10.3389/fimmu.2026.1898409

**Published:** 2026-10-29

**Authors:** Huang Chen, Ruiying Jiang, Yuhan Wu, Bin Tong, Lichao Jiang, Qixin Huang, Yao Liu, Qianqian Feng, Fang Yu, Rui Yang, Zhengyuan Guan, Ye Wang, Bei Wang, Dingrong Zhong, Meng Yang

**Affiliations:** 1 Department of Pathology, The China-Japan Friendship Hospital, Beijing, China; 2 National Center for Respiratory Medicine, State Key Laboratory of Respiratory Health and Multimorbidity, National Clinical Research Center for Respiratory Diseases, Institute of Respiratory Medicine, Chinese Academy of Medical Sciences, Department of Pulmonary and Critical Care Medicine, Center of Respiratory Medicine, China-Japan Friendship Hospital, Beijing, China

**Keywords:** CD4+/PD-1, CD8+/PD-1, dynamic biomarkers, lung cancer, PFS

## Abstract

**Background:**

Lung cancer is characterized by strong heterogeneity and dynamic evolution. Peripheral immune subsets and checkpoint molecules reflect tumor–immune interactions, but static biomarkers cannot capture temporal changes. This study assessed whether longitudinal trajectories of lymphocyte subsets and PD-1 expression could provide real-time risk stratification for progression-free survival (PFS).

**Methods:**

This retrospective cohort included 442 lung cancer patients with prospectively collected peripheral blood samples from an institutional biobank. Samples were obtained at baseline and multiple follow-ups. Frequencies of CD4^+^ T cells, CD8^+^ T cells, and natural killer (NK) cells, as well as PD-1 levels on CD4^+^ and CD8^+^ T cells, were measured by flow cytometry. Kaplan–Meier and time-dependent Cox models evaluated associations with PFS; discrimination was assessed via Harrell’s C-index.

**Results:**

Mean age was 63.1 ± 8.4 years; 76% were male, 77% had non-small cell lung cancer (NSCLC), and 81% presented at late stage. Progressive disease (PD) occurred in 13.1%. Longitudinal analysis revealed distinct immune alterations in PD patients. In single-immune-variable time-dependent Cox models, time-updated CD8^+^/PD-1 was an independent risk factor (HR = 9.40, 95% CI: 1.51–58.7, p = 0.017), while the other immune subsets were not significant in the overall population. Subgroup analyses showed consistent adverse effects of CD8^+^/PD-1 in NSCLC (HR = 545.5, p = 0.003), SCLC (HR = 666.0, p = 0.034), early-stage (HR = 2067.7, p = 0.015), and late-stage (HR = 248.8, p = 0.005) patients. Time-updated CD4^+^/PD-1 was protective in NSCLC (HR = 0.003, p = 0.009) and late-stage (HR = 0.005, p = 0.012). High baseline CD8^+^/PD-1 predicted shorter median PFS (p=0.020). The time-updated model showed good discrimination (C-index 0.694).

**Conclusion:**

Dynamic CD8^+^/PD-1 trajectories provide independent, adverse prognostic value, whereas preserved CD4^+^/PD-1 reflects immune competence in specific subgroups. Serial monitoring enables real-time risk assessment and personalized management in lung cancer.

## Introduction

Lung cancer remains the leading cause of cancer-related mortality worldwide, with non-small cell lung cancer (NSCLC) accounting for the majority of cases (1, 2). The advent of immune checkpoint inhibitors (ICIs), particularly those targeting the programmed death-1 (PD-1)/programmed death-ligand 1 (PD-L1) axis, has fundamentally transformed the therapeutic landscape, offering durable responses and improved survival for a subset of patients (3, 4). Despite these advances, clinical outcomes remain heterogeneous, with a significant proportion of patients exhibit primary resistance and others developing acquired resistance after an initial response (5, 6). Consequently, the identification of robust biomarkers to stratify risk and monitor therapeutic efficacy is an urgent clinical imperative (7).

Currently, clinical decision-making relies heavily on static, tissue-based biomarkers assessed at baseline, such as PD-L1 expression and tumor mutational burden (TMB) (8, 9). However, these static snapshots fail to capture the dynamic and evolving nature of the host-tumor immune interaction under therapeutic pressure (10). Recent evidence underscores the inadequacy of static assessments. In the neoadjuvant setting, Lin et al. demonstrated that traditional pathological response criteria, such as the percentage of residual viable tumor (%RVT), possess limited predictive power in stratifying outcomes within major pathologic response (MPR) subgroups (11). Notably, approximately 20% of patients achieving MPR or even complete pathologic response (pCR) still experience recurrence, highlighting a disconnection between static pathological endpoints and long-term survival (12, 13). Furthermore, radiological assessments based on RECIST criteria often fail to correlate with biological responses due to atypical patterns such as pseudoprogression, with studies showing misclassification rates as high as 41.5% for pathological responders (14, 15).

To overcome the limitations of static biomarkers, the focus has shifted toward dynamic monitoring. Liquid biopsy, specifically the longitudinal analysis of circulating tumor DNA (ctDNA), has emerged as a promising tool for detecting minimal residual disease (MRD) (16, 17). Qiu et al. demonstrated that serial ctDNA monitoring could anticipate radiological recurrence with a median lead time of 88 days and that joint modeling of longitudinal data significantly outperformed static landmark analyses in predicting recurrence risk (18). Similar evolutionary trajectories have been mapped in large-scale lung cancer cohorts, validating the utility of dynamic tracking (19). This paradigm shift, from a static to a dynamic approach, is equally pertinent to immunomonitoring. The systemic immune response, reflected in peripheral blood lymphocyte subsets, offers a minimally invasive window into the evolving tumor microenvironment (20).

Peripheral CD4^+^ T cells (CD3^+^CD4^+^), and CD8^+^ T cells (CD3^+^CD8^+^) cells, together with natural killer (NK, CD3^-^CD56^+^) cells, constitute the major circulating lymphocyte populations relevant to anti-tumor immunity. These populations were defined by surface marker expression (CD3^+^CD4^+^ and CD3^+^CD8^+^) rather than by functional assays; therefore, they represent phenotypically defined subsets that may include naive, memory, regulatory, and exhausted populations. Previous research in hepatocellular carcinoma has validated that the abundance and longitudinal changes of circulating CD4^+^ T cells and NK cells are superior predictors of objective response compared to static measures (21). In NSCLC, dynamic shifts in peripheral CD8^+^ T cell subsets have also been shown to correlate significantly with survival outcomes, reinforcing the cross-tumor applicability of this approach (22). Moreover, the expression of PD-1 on T cells is not merely a marker of exhaustion but a dynamic indicator of activation that fluctuates during therapy (23). Studies indicate that a proliferative burst of PD-1^+^ CD8^+^ T cells in peripheral blood often precedes clinical response, distinguishing responders from non-responders early in the treatment course (24, 25). Therefore, capturing the temporal trajectories of these immune parameters may provide critical prognostic information that baseline measurements miss.

Methodologically, standard survival analyses (e.g., baseline Cox models) are ill-suited for evaluating longitudinal biomarkers, as they ignore the time-varying nature of the data and may introduce immortal time bias (26). In contrast, time-dependent Cox proportional hazards models allow for the continuous updating of covariate values, thereby accurately quantifying the instantaneous risk associated with evolving immune phenotypes (27).

In this study, we conducted a retrospective observational analysis of 442 patients to evaluate the prognostic value of dynamic peripheral immune monitoring. We employed time-dependent Cox proportional hazards modeling—a statistical framework specifically designed for longitudinal data—to assess the association between the serial trajectories of circulating CD4^+^ T cells, CD8^+^ T cells, and NK cell subsets, as well as their PD-1 expression levels, and progression-free survival (PFS). Our objective was to move beyond static biomarker paradigms toward a dynamic, minimally invasive approach for precision risk stratification in the era of immuno-oncology.

## Materials and methods

### Patients

This retrospective observational cohort study was conducted at the China-Japan Friendship Hospital (CJFH) between July 2018 and October 2024. Peripheral blood samples were prospectively collected as part of routine clinical care and biobanking protocols established at our institution since 2018. For this retrospective study, patients were identified from the biobank database, and clinical outcomes were extracted from medical records. A total of 442 patients with pathologically confirmed lung cancer were reviewed and enrolled. Key inclusion criteria were: (1) adults (age ≥18 years); (2) pathologically confirmed primary lung cancer; (3) provision of written informed consent. Exclusion criteria included: (1) autoimmune diseases or active infections; (2) complicated with other malignancies; (3) administration of systemic immunosuppressive therapy at baseline. This study was approved by the Ethics Committee of CJFH (KY2023-169) and performed in accordance with the Declaration of Helsinki.

### Clinical data and response assessment

Baseline demographic and clinical information, including age, sex, and smoking history, was collected for all participants. Tumor characteristics were categorized by histology into NSCLC (including adenocarcinoma, squamous cell carcinoma, and others) and small cell lung cancer (SCLC). TNM staging was defined according to the AJCC 8^th^ edition. Progressive disease (PD) was defined as the first documentation of (1) radiological progression per RECIST v1.1 via scheduled imaging every 8–12 weeks, or (2) clinical progression documented by the treating physician. Of the 442 patients, 418 (94.6%) had at least one post-baseline radiological assessment. Among the 58 patients classified as having PD, all were based on documented disease progression (radiological per RECIST v1.1 or clinical); no patient without documented progression died within the surveillance window. The primary outcome, PFS, was defined as the interval from enrollment to the first documentation of objective radiographic or clinical progression or death from any cause. Patients were further stratified by surgery status into Early Stage (Surgery) and Late Stage (No Surgery) to control for the impact of therapeutic intervention. Treatment type was categorized as chemo-immunotherapy, chemotherapy alone, immunotherapy alone, targeted therapy, or other.

### Collection of peripheral blood and flow cytometry analysis

Peripheral blood samples were collected at baseline (Visit 1) and subsequent follow-up intervals (Visits 2-9) according to a predefined surveillance schedule. Baseline samples (Visit 1) were obtained prior to the initiation of any systemic therapy (chemotherapy, targeted therapy, or immunotherapy) and prior to surgical resection. For patients receiving systemic therapy, all follow-up samples were consistently collected immediately before the next scheduled treatment cycle (trough sampling) to minimize acute treatment-related fluctuations in immune parameters. For patients who underwent surgery, baseline samples were collected pre-operatively, and follow-up samples were obtained during post-operative surveillance visits. Samples were processed using standardized flow cytometry protocols to quantify absolute counts and frequencies of lymphocyte subsets. The following subsets were quantified: CD4^+^ T cells (CD3^+^CD4^+^), CD8^+^ T cells (CD3^+^CD8^+^), and NK cells (CD3^−^CD56^+^). Additionally, PD-1 expression was measured on CD4^+^ and CD8^+^ T cells populations. These subsets were defined solely by surface marker expression, and no functional assays (e.g., cytokine production, cytotoxicity) were performed. Therefore, the CD4^+^ and CD8^+^ populations should be interpreted as phenotypically defined subsets rather than functionally characterized helper or cytotoxic cells. Time-dependent variables of interest for analysis included CD4^+^ T cells% (percentage of lymphocytes that are CD4^+^ T cells), CD8^+^ T cells% (percentage of lymphocytes that are CD8^+^ T cells), NK% (percentage of lymphocytes that are NK), CD4^+^/PD-1% (percentage of CD4^+^ T cells expressing PD-1), and CD8^+^/PD-1% (percentage of CD8^+^ T cells expressing PD-1). The primary immunophenotypic endpoints focused on the dynamic trajectories of these markers over time.

Peripheral blood samples were processed within 4 hours of collection. Red blood cells were lysed using ammonium-chloride-potassium (ACK) lysis buffer (Thermo Fisher Scientific), and cells were washed twice with phosphate-buffered saline (PBS) containing 2% fetal bovine serum (FBS) and 0.1% sodium azide. Cell viability was assessed using trypan blue exclusion; samples with viability below 80% were excluded. The following antibodies were used for surface staining: anti-CD3-FITC (clone SK7, BD Biosciences), anti-CD4-PerCP-Cy5.5 (clone RPA-T4, BioLegend), anti-CD8-PE (clone RPA-T8, BD Biosciences), anti-CD56-APC (clone HCD56, BioLegend), and anti-PD-1-PE-Cy7 (clone EH12.2H7, BioLegend). Isotype-matched control antibodies (mouse IgG1, IgG2a) were used for each fluorochrome to establish non-specific binding thresholds. For each sample, 100 µL of cell suspension (approximately 1 × 10^5^ cells) was incubated with the antibody cocktail for 30 minutes at 4 °C in the dark. Following incubation, cells were washed twice with staining buffer (PBS with 2% FBS and 0.1% sodium azide) and fixed with 1% paraformaldehyde. Fluorescence-minus-one (FMO) controls were used for each fluorochrome to define positive gate boundaries for PD-1 expression.

Data were acquired on a BD FACSCanto II flow cytometer (BD Biosciences) using BD FACSDiva software (version 8.0). A minimum of 10,000 events were acquired per sample, with at least 100 events in the parent CD3^+^ T-cell population before subset analysis. All samples were evaluated individually for each patient; no sample pooling was performed. Samples with inadequate event counts (<10,000 total events or <100 CD3^+^ events) were excluded. Instrument performance was monitored daily using Cytometer Setup and Tracking (CS&T) beads (BD Biosciences) to ensure consistent fluorescence intensity across runs.

The gating strategy was as follows: (1) lymphocytes were identified by forward scatter (FSC) vs side scatter (SSC) characteristics; (2) singlet cells were discriminated using FSC-area (FSC-A) vs FSC-height (FSC-H); (3) CD3^+^ T cells were selected from the lymphocyte population; (4) CD4^+^ T cells and CD8^+^ T cells subsets were identified from the CD3^+^ population; (5) NK cells (CD3^−^CD56^+^) were identified from the lymphocyte population; and (6) PD-1 expression was measured on both CD4^+^ and CD8^+^ subsets as the percentage of positive cells within each subset using FMO-derived gates. Data were analyzed using FlowJo software (version 10.8, TreeStar).

### Statistical analysis

Continuous variables were summarized as mean ± standard deviation (SD) or median with interquartile range (IQR), and categorical variables were expressed as counts and percentages. Baseline differences between patients with PD and non-progressors (No PD) were assessed using t-tests, Wilcoxon rank-sum tests, Pearson’s Chi-squared tests, or Fisher’s exact tests as appropriate. For descriptive visualization of longitudinal trends, Locally Estimated Scatterplot Smoothing (LOESS) was applied to characterize dynamic trajectories of immune markers over time (in months). To formally assess whether trajectory patterns differed statistically between outcome groups, linear mixed-effects models were employed with fixed effects for time (months), outcome group (PD vs no PD), and their interaction, incorporating random intercepts for each patient to account for within-subject correlations. The significance of the group × time interaction was evaluated using likelihood ratio tests comparing models with and without the interaction term. Between-group comparisons at individual visits were performed using Wilcoxon rank-sum tests. Given the decreasing number of patients at risk at later timepoints, results from later visits were interpreted with appropriate caution.

For the primary analysis, time-dependent Cox proportional hazards models were utilized to assess the association between dynamic immunophenotypic alterations and PFS (27). To account for time-varying covariates, each patient’s follow-up period was segmented into consecutive risk intervals via a counting process data structure, which was defined by a start time t_(start)_ and a stop time _t(stop)_. Within each interval, the progression hazard was estimated using the most recently measured absolute levels of immune markers (e.g., CD4^+^/PD-1 and CD8^+^/PD-1), which were updated at the latest follow-up visit. To avoid collinearity arising from mathematically interdependent immune variables (e.g., CD8^+^ T cells, CD8^+^/PD-1, and ratio measures), each immune variable was tested in a separate time-dependent Cox model including all clinical covariates. To evaluate the incremental prognostic value of dynamic immune biomarkers beyond conventional clinical factors, nested Cox proportional hazards models were compared. Model 1 included only clinical covariates (age, sex, histology, surgical status, and TNM stage). Model 2 included clinical covariates plus baseline immune biomarkers (CD4^+^/PD-1, CD8^+^/PD-1, NK%, CD4^+^ T cells%, CD8^+^ T cells%, and CD4^+^-PD1/CD8^+^-PD1 ratio). Model 3 included clinical covariates plus time-updated immune biomarkers. Model discrimination was assessed using Harrell’s C-index with 95% confidence intervals estimated via 1,000 bootstrap resamples. Improvement in model performance was evaluated using the likelihood ratio test and the difference in C-index (ΔC-index) between nested models. Multicollinearity was assessed via the Variance Inflation Factor (VIF). Survival curves were estimated using the Kaplan–Meier method and compared using the log-rank test. For subgroup analyses, patients were stratified into NSCLC and SCLC, early-stage and late-stage, and within NSCLC by stage (I–III vs IV) according to the median value of each immune marker. A sensitivity analysis additionally adjusted for treatment type (chemo-immunotherapy, chemotherapy alone, immunotherapy alone, targeted therapy, or other). All statistical analyses were conducted using R software (version 4.3.0), with two-sided *P*-values < 0.05 considered statistically significant.

## Results

### Baseline characteristics

A total of 442 patients with pathologically confirmed lung cancer were enrolled in this retrospective cohort. As summarized in **Table 1**, the cohort was predominantly comprised of NSCLC (77%). The relatively low proportion of patients with documented disease progression (13.1%) reflects the limited median follow-up duration of 7.3 months (range: 0.4–47.2 months). No statistically significant differences were observed between the PD (n=58) and No PD (n=384) groups regarding sex (p=0.318) or histology (p=0.645). However, significant disparities were noted in age (60.5 ± 8.4 vs 63.3 ± 8.4 years, p=0.021), surgery status (p=0.009), and TNM stage distribution (p=0.006); specifically, Stage IV disease accounted for a significantly higher proportion of patients in the PD group (72%) compared to the No PD group (53%). The molecular landscape, including distribution of driver genes (e.g., EGFR, ALK, KRAS) and PD-L1 expression status, is detailed in **Supplementary Table 1**.

**Table 1:** Basic characteristics.

Characteristic	Overall (N = 442)	No PD (N = 384)	PD (N = 58)	p-value
Age (years), mean ± SD	63.1 ± 8.4	63.3 ± 8.4	60.5 ± 8.4	0.021
Sex, n (%)				0.318
Female	110 (25%)	92 (24%)	18 (31%)	
Male	332 (75%)	292 (76%)	40 (69%)	
Histology, n (%)				0.645
NSCLC	344 (78%)	297 (77%)	47 (81%)	
SCLC	98 (22%)	87 (23%)	11 (19%)	
Surgery status, n (%)				0.009
Early (surgery)	196 (44%)	180 (47%)	16 (28%)	
Late (no surgery)	246 (56%)	204 (53%)	42 (72%)	
TNM stage, n (%)				0.006
I–II	63 (14%)	61 (16%)	2 (3.4%)	
III	133 (30%)	119 (31%)	14 (24%)	
IV	246 (56%)	204 (53%)	42 (72%)	

PD, progressive disease; SD, standard deviation; NSCLC, non-small cell lung cancer; SCLC, small cell lung cancer; TNM, tumor-node-metastasis.

p-values from Student’s t-test (age), Pearson’s chi-square (sex, histology, surgery), or Fisher’s exact test (TNM).

### Longitudinal trends of immune markers

Longitudinal trajectories of peripheral immune markers were characterized using LOESS regression. **Figure 1** presents the complete set of all seven immune parameters in the overall cohort, stratified by outcome group (PD vs No PD). A distinct divergence in T-cell immunophenotypes was observed between outcome groups over time. In the PD group, CD8^+^/PD-1 levels exhibited a sustained upward trajectory, whereas CD4^+^/PD-1 levels demonstrated a relative decline or remained suppressed throughout follow-up. The CD4^+^-PD1/CD8^+^-PD1 ratio was consistently lower in the PD group, suggesting a progressive imbalance toward a more exhausted-like immunophenotype. NK cell counts showed a more rapid decline in the PD group compared to No PD patients. To formally evaluate whether the observed trajectory divergences were statistically significant, we performed linear mixed-effects modeling. CD8^+^/PD-1 demonstrated a borderline group × time interaction (p = 0.088), CD4^+^/PD-1 also showed a borderline group × time interaction (p = 0.085), and NK cell counts showed a borderline group × time interaction (p = 0.092) (**Supplementary Table 2**).

**Figure 1:**
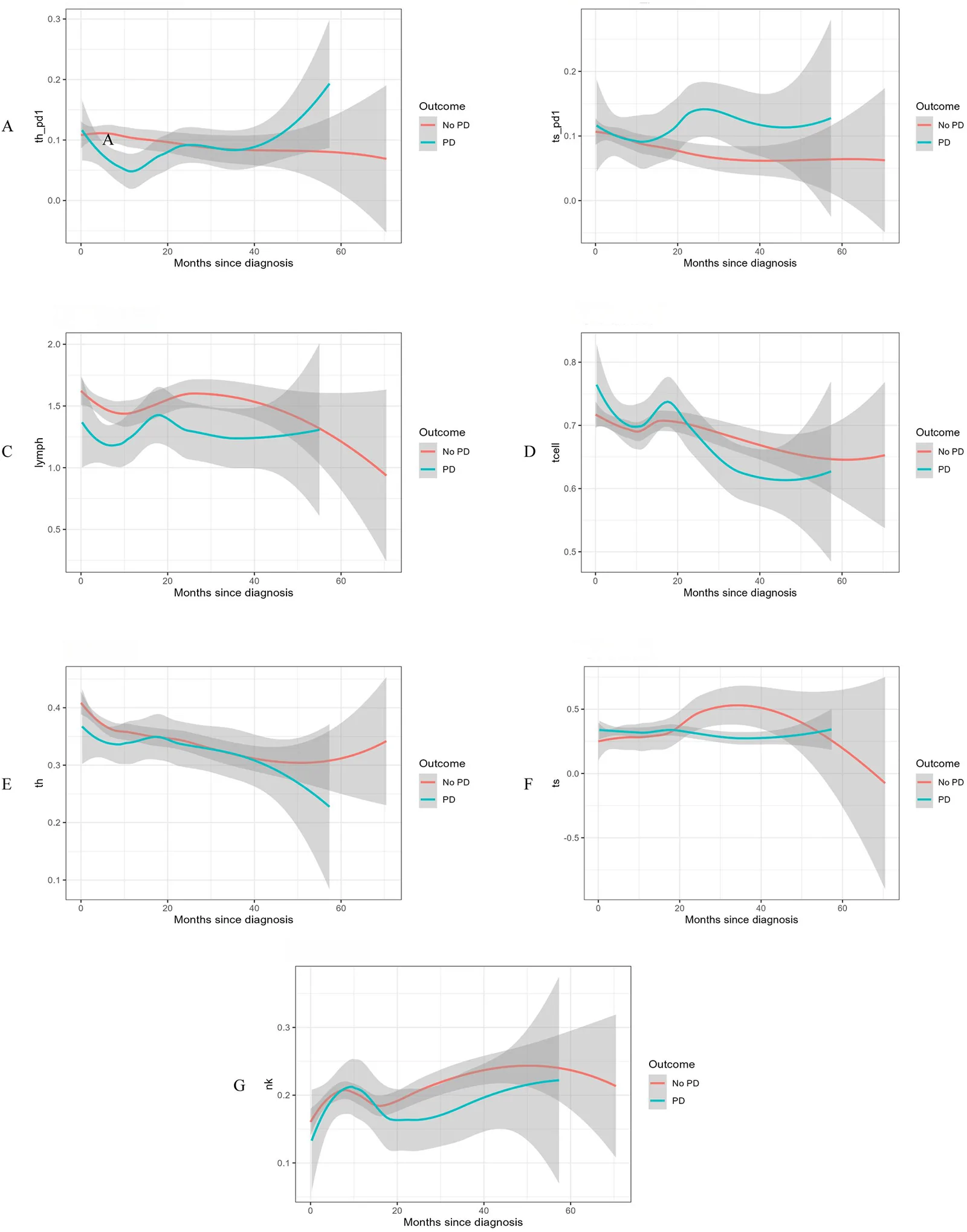
Longitudinal trends of immune cell levels for overall patients. Smoothed longitudinal trends (LOESS) of immune marker levels over time since diagnosis (months) in all patients, stratified by clinical outcome (No PD: red line; PD: blue line). Shaded areas represent 95% confidence intervals. Numbers of patients contributing samples at each visit: Visit 1 = 428, Visit 2 = 244, Visit 3 = 155, Visit 4 = 102, Visit 5 = 70, Visit 6 = 40, Visit 7 = 31, Visit 8 = 18, Visit 9 = 11. **(A)** CD4^+^-PD-1; **(B)** CD8^+^-PD1; **(C)** Lymphocyte count; **(D)** T cell count; **(E)** CD4^+^ T cells; **(F)** CD8^+^ T cells; and **(G)** NK cell.

When stratified by histology, distinct subtype-specific immunodynamic patterns emerged. In patients with NSCLC (**Figure 2**), PD patients experienced an early reduction in lymphocyte levels followed by a slow, partial recovery, with PD-1 expression on T cells rising gradually and continuously over the observation period. In contrast, patients with SCLC (**Figure 3**) showed rapid and dramatic immunological changes: lymphocyte, NK cell, and T cell counts dropped sharply within the first 20 months post-diagnosis, and PD-1 expression on helper T cells showed a transient early increase. This aggressive immune erosion aligns with the rapid clinical progression of SCLC.

**Figure 2:**
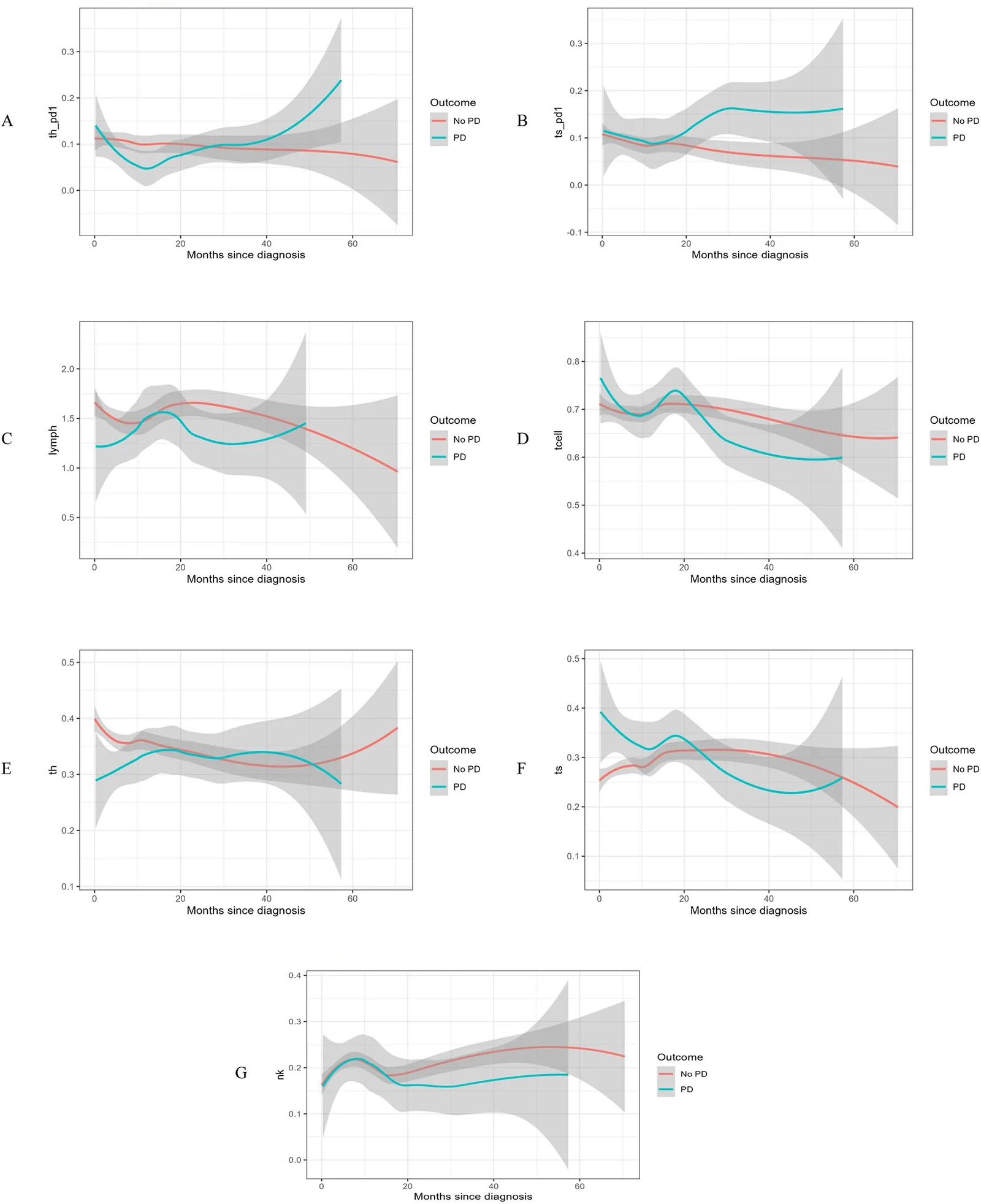
Longitudinal trends of immune cell levels for NSCLC patients. Smoothed longitudinal trends (LOESS) of immune marker levels over time since diagnosis (months) in patients with NSCLC, stratified by clinical outcome (No PD: blue line; PD: yellow line). Shaded areas represent 95% confidence intervals. **(A)** CD4^+^-PD-1; **(B)** CD8^+^-PD1; **(C)** Lymphocyte count; **(D)** T cell count; **(E)** CD4^+^ T cells; **(F)** CD8^+^ T cells; and **(G)** NK cell.

**Figure 3:**
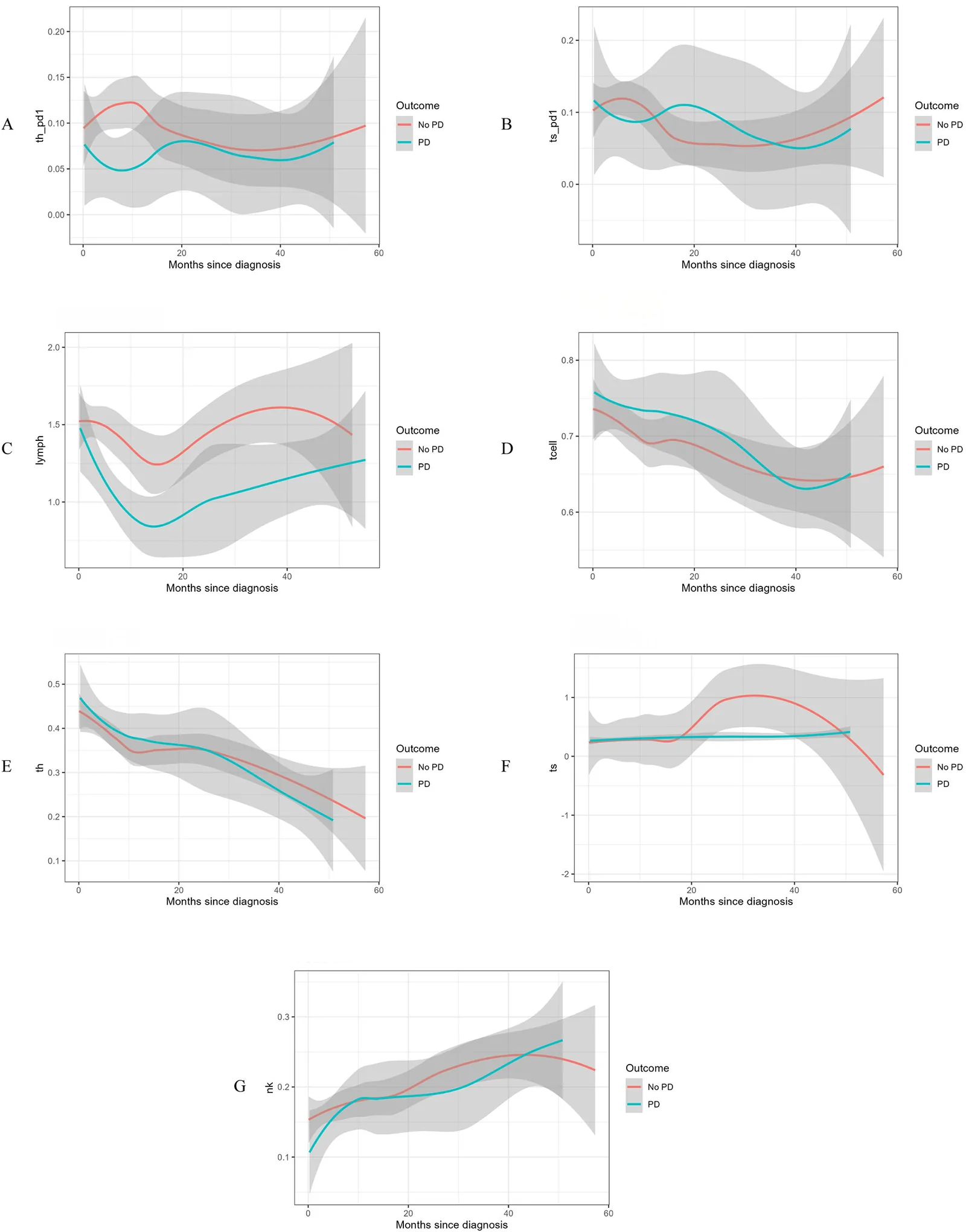
Longitudinal trends of immune cell levels for SCLC patients. Smoothed longitudinal trends (LOESS) of immune marker levels over time since diagnosis (months) in patients with SCLC, stratified by clinical outcome (No PD: blue line; PD: yellow line). Shaded areas represent 95% confidence intervals. **(A)** CD4^+^-PD-1; **(B)** CD8^+^-PD1; **(C)** Lymphocyte count; **(D)** T cell count; **(E)** CD4^+^ T cells; **(F)** CD8^+^ T cells; and **(G)** NK cell.

Stratification by surgery status revealed that early-stage patients maintained relatively stable immune parameters over time (**Supplementary Figure 1**), whereas late-stage patients exhibited more pronounced immunologic deterioration, particularly in NK cell counts and CD8^+^/PD-1 expression (**Supplementary Figure 2**). These findings suggest that tumor burden (reflected by stage and surgical resection) may modulate the rate of immune decline. Visit-level comparisons of immune cell levels at each follow-up are provided in **Supplementary Table 3**.

### Association with progression-free survival

The prognostic value of dynamic immune markers was evaluated using time-dependent Cox proportional hazards model. To avoid collinearity arising from mathematically interdependent immune variables (e.g., CD8^+^ T cells, CD8^+^/PD-1, and ratio measures), each immune variable was tested in a separate time-dependent Cox model including all clinical covariates. In the overall population, only time-updated CD8^+^/PD-1 was a significant independent risk factor for progression (HR = 9.40, 95% CI: 1.51–58.7, p = 0.017); the other immune variables did not reach statistical significance (**Table 2**).

**Table 2:** Single-immune-variable time-dependent Cox models (overall population).

Immune variable	HR	95% CI	p-value
Lymph	0.70	0.48–1.02	0.063
T cell	1.26	0.26–6.05	0.776
CD4^+^ T cells	0.39	0.06–2.51	0.321
CD8^+^ T cells	5.09	0.87–30.0	0.072
CD4^+^/PD-1	0.81	0.11–6.07	0.834
CD8^+^/PD-1	9.40	1.51–58.7	0.017
NK	0.45	0.05–4.02	0.477

Each immune variable was tested in a separate time-dependent Cox model including age, sex, TNM stage, histology, and surgery. Immune variables were not entered simultaneously to avoid collinearity.

Subgroup analyses (**Table 3**) showed consistent adverse effects of CD8^+^/PD-1 across all subgroups: NSCLC (HR = 545.5, 95% CI: 8.30–35868, p = 0.003), SCLC (HR = 666.0, 95% CI: 1.63–271878, p = 0.034), early-stage (HR = 2067.7, 95% CI: 4.53–944134, p = 0.015), late-stage (HR = 248.8, 95% CI: 5.15–12028, p = 0.005), and NSCLC stage I–III (HR = 1.08×10^6^, 95% CI: 160–7.2×10^9^, p = 0.002). Time-updated CD4^+^/PD-1 was protective in NSCLC (HR = 0.003, 95% CI: 0.00004–0.238, p = 0.009) and late-stage patients (HR = 0.005, 95% CI: 0.00008–0.306, p = 0.012).

**Table 3:** Subgroup analyses (time-dependent Cox).

Subgroup	n	Events	CD8^+^/PD-1 HR (95% CI)	P	CD4^+^/PD-1 HR (95% CI)	p
NSCLC	681	54	545.5 (8.30–35868)	0.003	0.003 (0.00004–0.238)	0.009
SCLC	243	17	666.0 (1.63–271878)	0.034	0.033 (0.00005–24.0)	0.311
Early (surgery)	393	19	2067.7 (4.53–944134)	0.015	0.012 (0.00002–7.28)	0.175
Late (no surgery)	531	52	248.8 (5.15–12028)	0.005	0.005 (0.00008–0.306)	0.012
NSCLC I–III	315	12	1.08×10^6^ (160–7.2×10^9^)	0.002	0.0002 (2×10^−8^–1.49)	0.061
NSCLC IV	366	42	100.5 (0.56–18062)	0.082	0.006 (0.00004–1.14)	0.056

NSCLC, non-small cell lung cancer; SCLC, small cell lung cancer.

To evaluate whether dynamic immune biomarkers provide incremental prognostic value beyond established clinical factors, we compared nested Cox models (**Table 4**, Panel A). The clinical model achieved a C-index of 0.637; addition of baseline immune biomarkers increased it to 0.687; the time-updated immune model achieved a C-index of 0.694. The likelihood ratio test comparing the clinical and baseline models was borderline significant (χ² = 7.75, df = 3, p = 0.052).

**Table 4:** Model comparison and treatment-adjusted model.

Panel A. Nested model comparison			
Model		C-index	
Clinical only		0.637	
Baseline + clinical		0.687	
Time-updated + clinical		0.694	

Panel B. Treatment-adjusted time-dependent Cox model			
Variable	HR	95% CI	p
Age	0.97	0.95–1.00	0.045
Sex (male)	2.74	1.58–4.76	0.0004
TNM III	4.66	1.06–20.4	0.041
TNM IV	9.05	2.13–38.5	0.003
Chemotherapy	2.26	1.20–4.25	0.011
Immunotherapy	0.55	0.29–1.03	0.064
Targeted	1.85	0.97–3.55	0.063
CD4^+^/PD-1	0.001	0.00003–0.034	0.0001
CD8^+^/PD-1	1615.7	85.9–30372	<0.0001

Likelihood ratio test (Model 1 vs Model 2): χ² = 7.75, df = 3, p = 0.052.

A sensitivity analysis adjusting for treatment type confirmed that time-updated CD8^+^/PD-1 remained a strong independent risk factor (HR = 1615.7, 95% CI: 85.9–30372, p < 0.0001) and CD4^+^/PD-1 remained protective (HR = 0.001, 95% CI: 0.00003–0.034, p = 0.0001) (**Table 4**, Panel B).

### Baseline prognostic value

Kaplan–Meier survival analyses were conducted to assess the impact of baseline immune markers on long-term outcomes. The overall PFS curve is shown in **Figure 4A**. When patients were stratified by TNM stage, a clear trend toward shorter PFS with advanced disease stage was observed (p = 0.037, **Figure 4B**). When stratified by histology, NSCLC patients had a median PFS of 49.12 months (95% CI: 35.45–NR), whereas SCLC patients did not reach median PFS (**Figure 5A**). When stratified by surgery status, early-stage patients had a median PFS of 49.12 months (95% CI: 40.77–NR), and late-stage patients had a median PFS of 56.87 months (95% CI: 32.33–NR) (**Figure 5B**). The combination of histology and surgery status is shown in **Supplementary Figure 3**.

**Figure 4:**
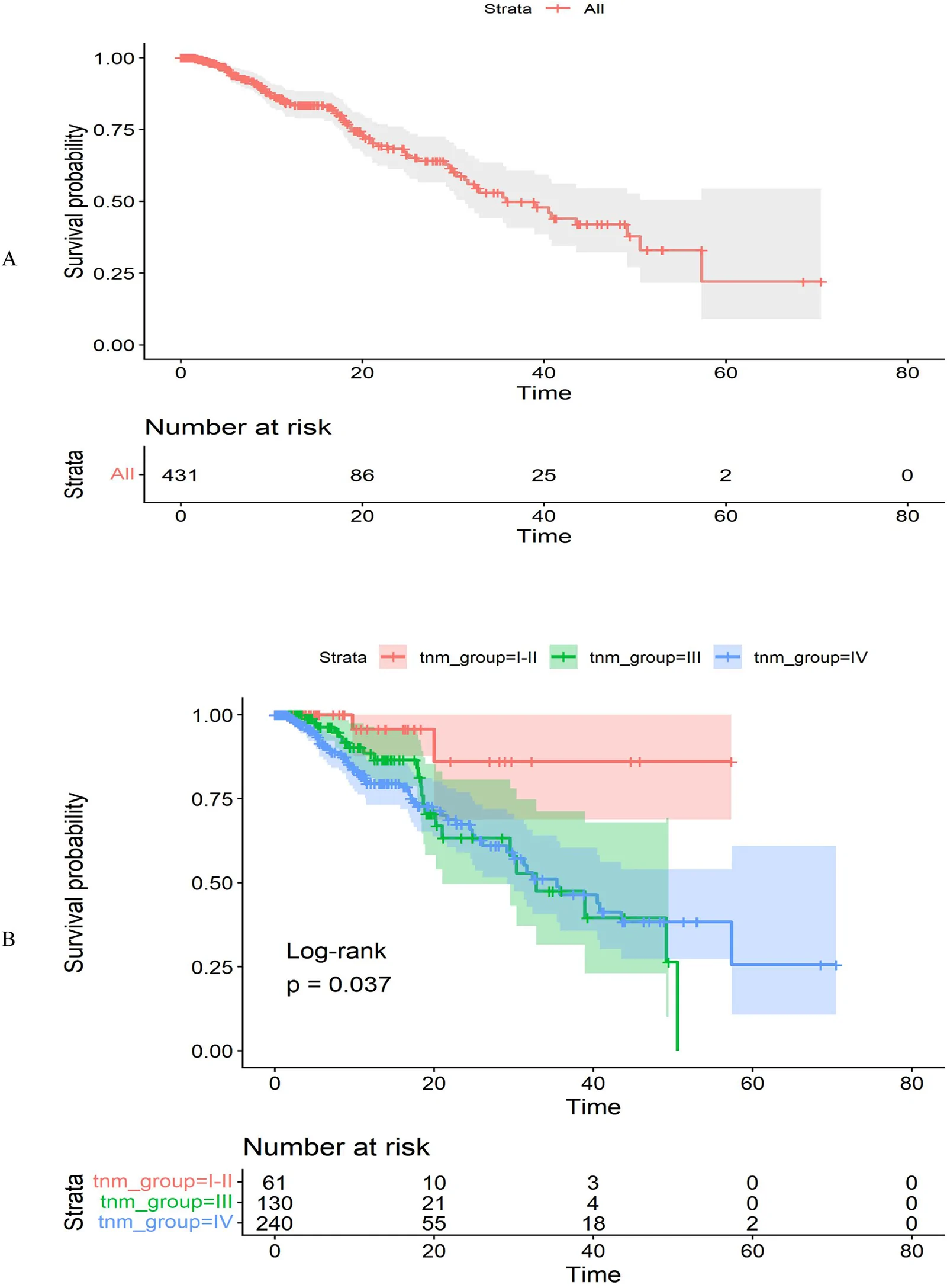
Kaplan-Meier analysis of progression-free survival. Kaplan–Meier curves showing PFS in all patients **(A)** and stratified by TNM stage **(B)**. Shaded areas represent 95% confidence intervals. Number at risk shown below each panel at 20-month intervals.

**Figure 5:**
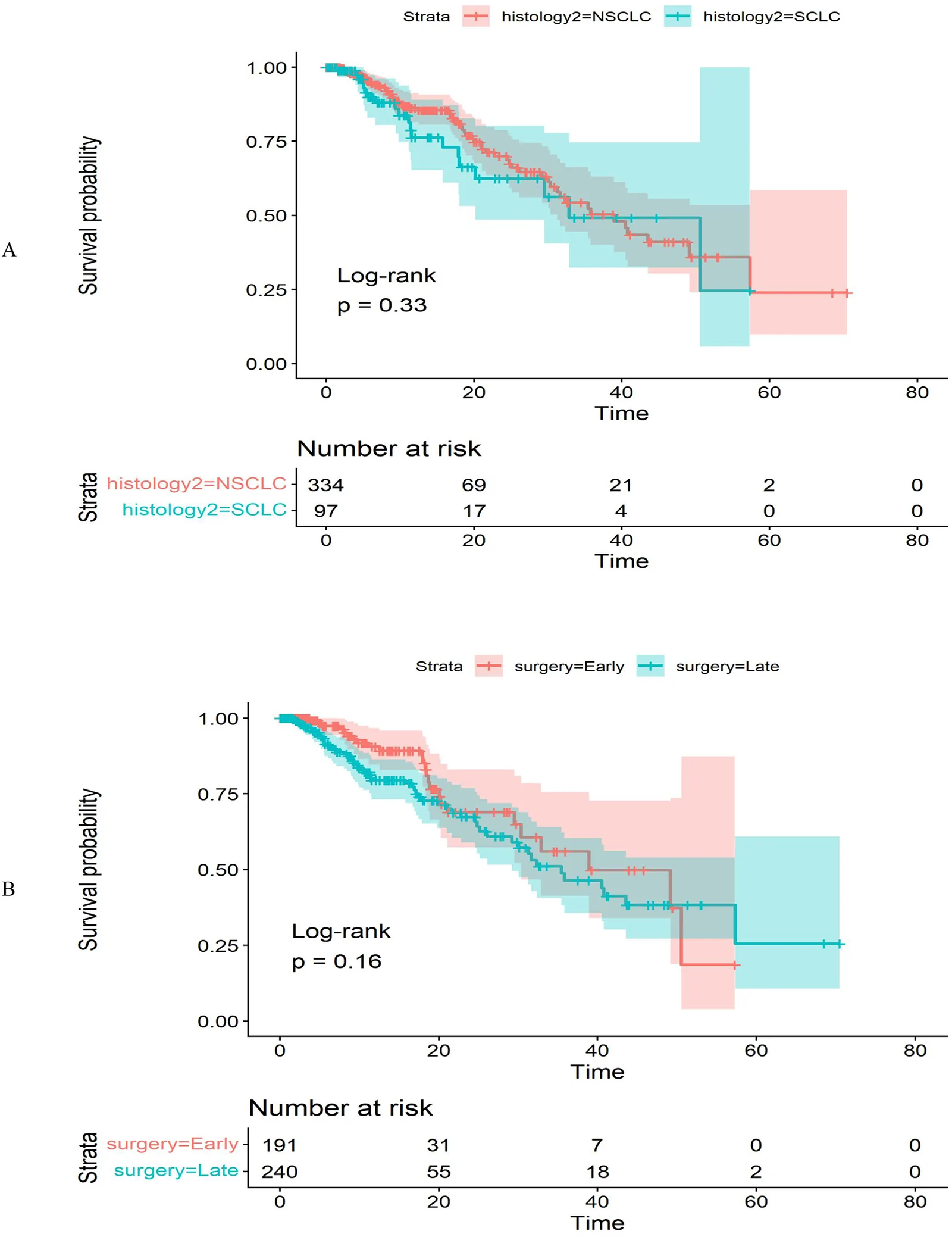
Kaplan-Meier analysis of progression-free survival by histology and surgery. Kaplan–Meier curves showing PFS stratified by histology **(A)** and surgery status **(B)**. Shaded areas represent 95% confidence intervals.

High baseline expression of CD8^+^/PD-1 was significantly associated with a shorter median PFS compared to the low expression group (40.77 months vs. 141.73 months; p = 0.020, **Figure 6A**). Analysis of the baseline CD4^+^-PD1/CD8^+^-PD1 ratio further corroborated these findings, with the low-ratio group demonstrating a significantly shorter median PFS of 31.64 months compared to 141.73 months in the high-ratio group (p < 0.001, **Figure 6B**). Baseline lymphocyte, T cell, and NK cell levels are shown in **Supplementary Figures 4**–**6**, respectively.

**Figure 6:**
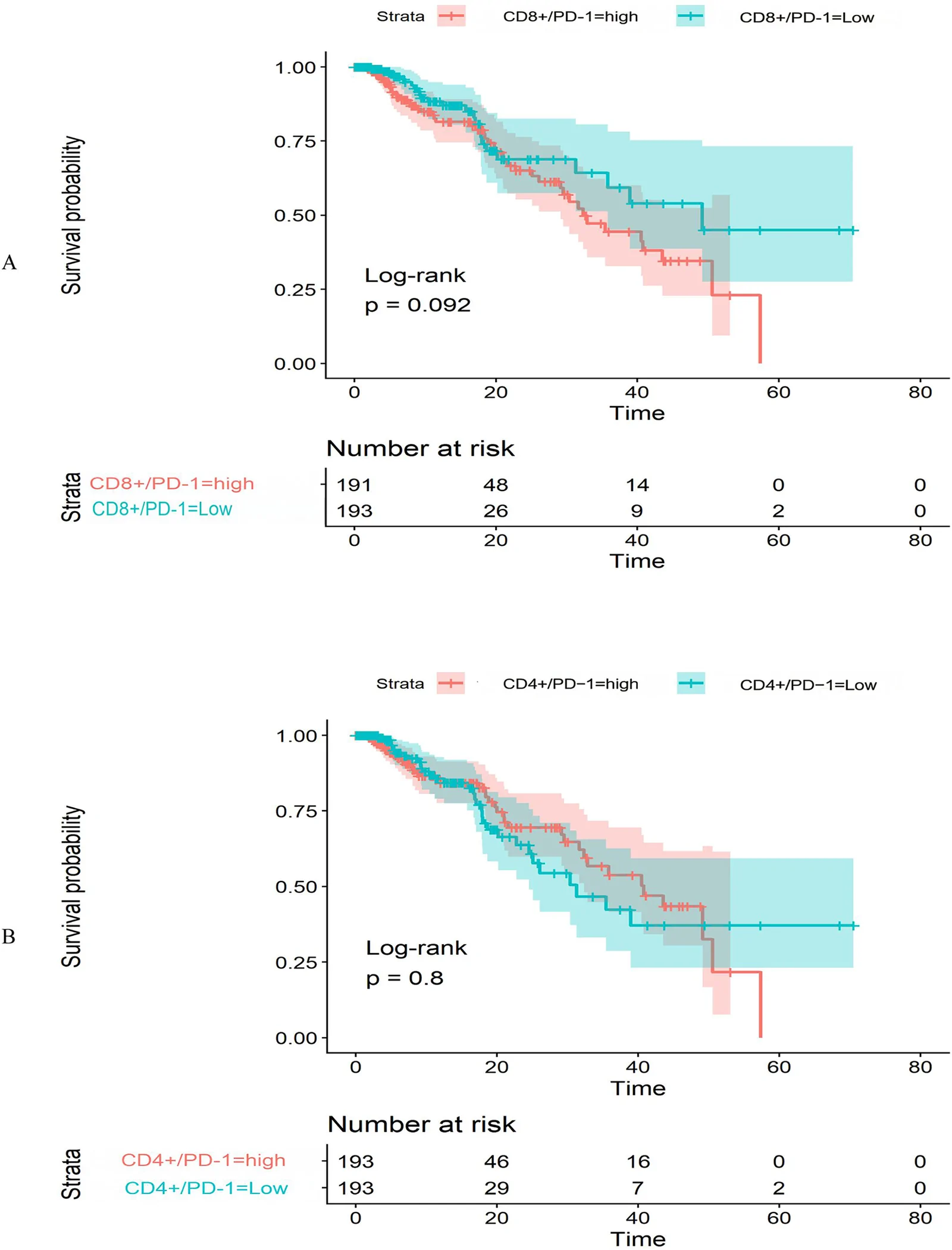
Kaplan-Meier analysis of progression-free survival by baseline immune markers. Kaplan–Meier curves showing PFS stratified by baseline CD8^+^/PD-1 **(A)** and baseline CD4^+^-PD1/CD8^+^-PD1 ratio **(B)**. Shaded areas represent 95% confidence intervals.

## Discussion

This study demonstrates that dynamic monitoring of peripheral immune phenotypes, particularly CD4^+^/PD-1 and CD8^+^/PD-1, provides independent prognostic information for progression risk in patients with lung cancer. In the analysis, only time-updated CD8^+^/PD-1 remained an independent adverse risk factor in the overall population, with consistent effects across NSCLC, SCLC, early-stage, and late-stage subgroups. Time-updated CD4^+^/PD-1 was protective in NSCLC and late-stage patients. This divergent pattern was observed within the same patients over time and confirmed across histology- and stage-specific subgroups, suggesting that PD-1 expression on helper T cells may reflect sustained immune competence, while PD-1 on cytotoxic T cells signals terminal dysfunction. In addition, the time-updated immune model showed better discrimination than both the clinical model and the baseline-immune model, establishing a framework for real-time risk reassessment that could inform clinical decision-making.

The methodological approach of testing each immune variable in a separate time-dependent Cox model was adopted to avoid collinearity arising from mathematically interdependent variables (e.g., CD8^+^ T cells, CD8^+^/PD-1, and ratio measures). This approach yielded stable and interpretable estimates, in contrast to the initial full model in which extreme HRs and confidence intervals were observed. The independent contribution of each immune variable could thus be evaluated against the same set of clinical covariates, ensuring that the reported associations were not driven by statistical artifacts.

The mechanistic basis for these opposing trajectories likely resides in the functional dichotomy of PD-1 signaling across distinct T-cell compartments. Consistent with the concept of “T-cell exhaustion,” the sustained elevation of CD8^+^/PD-1 expression observed in our progressing cohort likely is consistent with, though not definitive for, an exhausted-like T-cell state. While PD-1 expression is a hallmark of T-cell exhaustion, definitive characterization requires assessment of additional markers such as TIM-3, LAG-3, and TCF-1, as well as functional assays. Our data demonstrate an association between elevated CD8^+^/PD-1 and adverse outcomes, which may reflect immunologic dysfunction, but we acknowledge that PD-1 alone cannot definitively establish exhaustion (28, 29). In sharp contrast, the protective nature of time-updated CD4^+^/PD-1 expression suggests that PD-1 on helper T cells may not solely represent exhaustion but rather serve as a marker of “Immune Competence” or antigen-experienced activation required for an effective antitumor response (30, 31). This bifurcation—where PD-1 signaling signifies potential activation in the helper subset but terminal exhaustion in the cytotoxic subset—highlights the critical necessity of considering the cellular context when interpreting PD-1 as a dynamic biomarker (32, 33).

It is important to note that the CD4^+^ and CD8^+^ T cell populations analyzed in this study were defined by surface marker expression (CD3^+^CD4^+^ and CD3^+^CD8^+^), rather than by functional characterization. Circulating CD4^+^ T cells include not only active helper cells but also naive cells and regulatory T cells, while CD8^+^ T cells include naive, central memory, effector memory, and exhausted populations. Therefore, the divergent prognostic roles of CD4^+^/PD-1 and CD8^+^/PD-1 observed here should be interpreted as associations with phenotypically defined subsets rather than with functionally distinct helper or cytotoxic populations. Future studies incorporating functional assays (e.g., cytokine production, cytotoxicity, proliferation) and additional phenotypic markers (e.g., TIM-3, LAG-3, TCF-1) will be necessary to fully characterize the biological state of these PD-1^+^ T cell populations.

The subgroup-specific patterns observed in our analysis further support this functional dichotomy. In NSCLC patients, both CD8^+^/PD-1 and CD4^+^/PD-1 were significant, suggesting that the balance between cytotoxic exhaustion and helper competence is particularly relevant in this histology. In SCLC, only CD8^+^/PD-1 was significant, consistent with the more aggressive immune erosion observed in this subtype. In early-stage patients who underwent surgery, CD8^+^/PD-1 was the dominant prognostic signal, whereas in late-stage patients both CD8^+^/PD-1 and CD4^+^/PD-1 contributed, consistent with the more pronounced immunologic deterioration observed in late-stage disease.

Several prior studies have investigated peripheral PD-1^+^ T-cell subsets in lung cancer. Ottonello et al. demonstrated that baseline CD8^+^PD-1^+^ T-cell frequencies correlated with response to nivolumab in NSCLC patients (22). Kamphorst et al. showed that proliferation of PD-1^+^ CD8^+^ T cells in peripheral blood preceded clinical response to PD-1-targeted therapy (24). However, these studies were limited to single or early timepoint assessments and did not capture the longitudinal evolution of immune phenotypes under clinical pressure. Our study extends these findings by demonstrating that serial monitoring, and time-dependent analysis can identify when immune phenotypes diverge between progressors and non-progressors—information that static assessments cannot provide. Additionally, the divergent prognostic roles of CD4^+^/PD-1 versus CD8^+^/PD-1 that we observed have not been previously reported in the context of longitudinal monitoring, to our knowledge, and may have implications for understanding the functional heterogeneity of PD-1 signaling across T-cell subsets.

Methodologically, the utilization of a time-dependent Cox model provides a substantial advantage over traditional landmark analyses by capturing “Real-time Risk” (27). This framework divides the follow-up period into discrete intervals, utilizing the most recent immunophenotypic data to update the hazard assessment for each patient. This approach parallels the clinical utility seen in longitudinal ctDNA monitoring, where serial measurements are used to dynamically predict recurrence and guide therapeutic adjustments (17, 18). The model’s robust discriminative performance, indicated by a Harrell’s C-index of 0.694, further validates the prognostic relevance of integrating dynamic peripheral biomarkers into routine lung cancer surveillance. A sensitivity analysis adjusting for treatment type confirmed that time-updated CD8^+^/PD-1 remained a strong independent risk factor and CD4^+^/PD-1 remained protective. This finding indicates that the divergent prognostic roles of CD8^+^/PD-1 and CD4^+^/PD-1 are not solely driven by treatment-related immune modulation and remain robust after adjustment for chemo-immunotherapy, chemotherapy alone, immunotherapy alone, and targeted therapy.

The incremental improvement in model discrimination (C-index from 0.637 to 0.694) upon addition of dynamic immune biomarkers has potential clinical implications. While the absolute improvement may appear modest, in the context of risk stratification for clinical decision-making, even a modest C-index improvement can translate into meaningful reclassification of patients, particularly when used to guide surveillance intensity or timing of therapeutic intervention. The nested model comparison also addresses a key limitation of prior studies that have reported associations between static immune markers and prognosis without demonstrating added value over established clinical variables. Our findings suggest that serial immunomonitoring could be particularly valuable in patients with intermediate-risk clinical profiles, where imaging findings are equivocal or where routine surveillance provides limited predictive information.

An important consideration regarding the generalizability of our findings is the predominantly male composition of our cohort (76%, n=334). This sex distribution reflects the higher incidence of lung cancer in males in China, where the male-to-female incidence ratio is approximately 2.3:1 according to national cancer registry data. Females generally exhibit stronger innate and adaptive immune responses, characterized by higher CD4^+^ T-cell counts, greater antibody production, and more robust type I interferon responses, whereas males tend to have higher baseline inflammatory cytokine levels and stronger myeloid-derived suppressor cell activity. These immunological differences could potentially influence the baseline levels and longitudinal trajectories of the peripheral immune markers we studied, as well as their prognostic value. To address the potential confounding effect of sex, we included sex as a covariate in all multivariable time-dependent Cox models. In the treatment-adjusted model, male sex was associated with increased progression risk. These findings are consistent with the known poorer prognosis of male lung cancer patients and suggest that sex contributes independently to prognostic risk beyond immune markers.

While the hazard ratio for CD8^+^/PD-1 is numerically large, this reflects the unit-scaling effect of analyzing a proportion (0–1) as a continuous variable rather than collinearity (VIF < 2). Effectively, this indicates that distinct upward trajectories in CD8^+^/PD-1 expression are strongly and independently associated with elevated progression risk, validating its utility for real-time risk stratification (34, 35). However, despite this inflation, the statistical significance and directionality of the risk association remained consistent across the overall population and histology-specific subgroups (e.g., NSCLC), so while the precise magnitude of the HR should be interpreted with caution, the qualitative conclusion that rising CD8^+^/PD-1 is a robust predictor of inferior PFS remains solid.

Several limitations of this study should be acknowledged. First, peripheral blood biomarkers may not fully capture tumor microenvironment dynamics, and correlations with tumor PD-L1 expression or tumor-infiltrating lymphocytes were not assessed; although a sensitivity analysis adjusting for treatment type was performed, residual confounding by concomitant anti-tumor therapies cannot be excluded. Second, we acknowledge that PD-1 expression alone is insufficient to define T-cell exhaustion definitively; future studies incorporating additional exhaustion markers (e.g., TIM-3, LAG-3, TCF-1) and functional assays will be necessary to fully characterize the biological state of PD-1^+^ T cells. Third, the predominantly male cohort (76%) may limit the generalizability of our findings. Fourth, the model was developed on a single retrospective cohort and has not been externally validated. Fifth, the restriction of PFS events to the first two post-baseline assessments, consistent with our predefined surveillance window, may underestimate long-term progression events. Sixth, the number of patients with samples at later follow-up visits declined substantially, which limited statistical power for visit-level comparisons beyond Visit 4. Seventh, the SCLC subgroup had a limited number of events (n = 17), and subgroup-specific results should be interpreted with caution. Finally, the CD4^+^ and CD8^+^ T cell populations analyzed here were defined by surface marker expression rather than by functional characterization; they may therefore include naive, memory, regulatory, and exhausted subsets, and our findings should be interpreted accordingly.

## Conclusion

In conclusion, this study demonstrates that longitudinal, rather than static, monitoring of peripheral CD4^+^/PD-1 and CD8^+^/PD-1 trajectories provides a non-invasive framework for real-time risk stratification in lung cancer. Our findings identify time-updated CD8^+^/PD-1 as an independent adverse risk factor, with consistent effects across NSCLC, SCLC, early-stage, and late-stage subgroups, while time-updated CD4^+^/PD-1 was protective in NSCLC and late-stage patients. The major contribution of this work is methodological (establishing the value of serial immunomonitoring with time-dependent analysis) and conceptual (demonstrating divergent prognostic roles for CD4^+^/PD-1 versus CD8^+^/PD-1 when assessed dynamically). These results support the clinical utility of longitudinal immunomonitoring for risk stratification and the potential to guide timely therapeutic interventions. Prospective validation in larger, treatment-stratified cohorts will be essential to confirm these findings.

## Data Availability

The original contributions presented in the study are included in the article/**Supplementary Material**. Further inquiries can be directed to the corresponding authors.

## Supplementary material

Supplementary Figure 1 Longitudinal Trends of Immune Cell Levels for Patients with Early Stage. Smoothed longitudinal trends (LOESS) of immune marker levels over time since diagnosis (months) in early-stage patients treated with surgery, stratified by clinical outcome (No PD: blue line; PD: yellow line). Shaded areas represent 95% confidence intervals. **(A)** CD4^+^-PD-1; **(B)** CD8^+^-PD1; **(C)** Lymphocyte count; **(D)** T cell count; **(E)** CD4^+^ T cells; **(F)** CD8^+^ T cells; and **(G)** NK cell.

Supplementary Figure 2 Longitudinal Trends of Immune Cell Levels for Patients with Late Stage. Smoothed longitudinal trends (LOESS) of immune marker levels over time since diagnosis (months) in late-stage patients without surgical treatment, stratified by clinical outcome (No PD: blue line; PD: yellow line). Shaded areas represent 95% confidence intervals. **(A)** CD4^+^-PD-1; **(B)** CD8^+^-PD1; **(C)** Lymphocyte count; **(D)** T cell count; **(E)** CD4^+^ T cells; **(F)** CD8^+^ T cells; and **(G)** NK cell.

Supplementary Figure 3 Kaplan-Meier Analysis of Progression-Free Survival by Histology and Stage Combination.

Supplementary Figure 4 Kaplan-Meier Analysis of Progression-Free Survival by Baseline Lymphocyte Level.

Supplementary Figure 5 Kaplan-Meier Analysis of Progression-Free Survival by Baseline T Cell Level.

Supplementary Figure 6 Kaplan-Meier Analysis of Progression-Free Survival by Baseline NK Cell Level.
