# Supplementary material for: Longitudinal trajectories of peripheral blood lymphocyte subsets and PD-1 expression as dynamic prognostic markers in lung cancer
Source: Front Immunol. 2026 Oct 29;17:1898409. doi: 10.3389/fimmu.2026.1898409 (PMC13755337; doi:10.3389/fimmu.2026.1898409)
Supplement: Supplementary file 1 [file Table1.docx]

# Supplementary Table 1. Distribution of Driver Gene and PD_L1 Status

| Characteristic | Overall (N=442) | No PD (N=384) | PD (N=58) | p-value |
| --- | --- | --- | --- | --- |
| Driver gene, n (%) |  |  |  | <0.001 |
| Negative | 48 (11%) | 41 (11%) | 7 (12%) |  |
| EGFR | 65 (15%) | 49 (13%) | 16 (28%) |  |
| KRAS | 27 (6.1%) | 25 (6.5%) | 2 (3.4%) |  |
| ALK | 10 (2.3%) | 5 (1.3%) | 5 (8.6%) |  |
| HER2 | 4 (0.9%) | 4 (1.0%) | 0 (0%) |  |
| MET | 3 (0.7%) | 2 (0.5%) | 1 (1.7%) |  |
| Others | 12 (2.7%) | 10 (2.6%) | 2 (3.4%) |  |
| Not Tested | 273 (62%) | 248 (65%) | 25 (43%) |  |
| PD-L1 status, n (%) |  |  |  | 0.015 |
| Negative | 160 (36%) | 131 (34%) | 29 (50%) |  |
| Positive | 23 (5.2%) | 18 (4.7%) | 5 (8.6%) |  |
| Not Tested | 259 (59%) | 235 (61%) | 24 (41%) |  |

Abbreviations: EGFR, epidermal growth factor receptor; ALK, anaplastic lymphoma kinase; KRAS, Kirsten rat sarcoma viral oncogene homolog; HER2, human epidermal growth factor receptor 2; MET, mesenchymal-epithelial transition factor; PD-L1, programmed death-ligand 1.
