# Supplementary material for: Longitudinal trajectories of peripheral blood lymphocyte subsets and PD-1 expression as dynamic prognostic markers in lung cancer
Source: Front Immunol. 2026 Oct 29;17:1898409. doi: 10.3389/fimmu.2026.1898409 (PMC13755337; doi:10.3389/fimmu.2026.1898409)
Supplement: Supplementary file 2 [file Table2.docx]

# Supplementary Table 2. Linear Mix-Effects Model Results

| Marker | Chisq | Df | p |
| --- | --- | --- | --- |
| lymph | 0.002 | 1 | 0.964 |
| T cell | 1.183 | 1 | 0.277 |
| CD4^+^ cell | 2.972 | 1 | 0.085 |
| CD8^+^ cell | 0.818 | 1 | 0.366 |
| CD4^+^-pd1 | 1.274 | 1 | 0.259 |
| CD8^+^-pd1 | 2.919 | 1 | 0.088 |
| nk | 2.834 | 1 | 0.092 |

Note: Group × time interaction tested by likelihood ratio test comparing models with and without the interaction term.
