# Supplementary material for: Longitudinal trajectories of peripheral blood lymphocyte subsets and PD-1 expression as dynamic prognostic markers in lung cancer
Source: Front Immunol. 2026 Oct 29;17:1898409. doi: 10.3389/fimmu.2026.1898409 (PMC13755337; doi:10.3389/fimmu.2026.1898409)
Supplement: Supplementary file 3 [file Table3.docx]

Supplementary Table 3. Immune Cell levels at Each Visit and Number of Patients Contributing Samples

Panel A. Number of patients at each visit

| Visit | n_total | No PD | PD |
| --- | --- | --- | --- |
| 1 | 428 | 372 | 56 |
| 2 | 244 | 213 | 31 |
| 3 | 155 | 145 | 10 |
| 4 | 102 | 95 | 7 |
| 5 | 70 | 65 | 5 |
| 6 | 40 | 37 | 3 |
| 7 | 31 | 28 | 3 |
| 8 | 18 | 17 | 1 |
| 9 | 11 | 10 | 1 |

Panel B. Visit-level immune cell levels (median, IQR)

| Visit | Variable | No PD | PD | p |
| --- | --- | --- | --- | --- |
| 1 | Lymph | 1.41 (1.06–1.87) | 1.21 (1.04–1.54) | 0.047 |
| 1 | CD4^+^ cell | 0.373 (0.30–0.45) | 0.335 (0.27–0.42) | 0.046 |
| 1 | CD8^+^/PD-1 | 0.045 (0.01–0.15) | 0.116 (0.05–0.19) | <0.001 |
| 1 | CD4^+^/PD-1 | 0.060 (0.03–0.12) | 0.078 (0.04–0.11) | 0.381 |
| 1 | NK | 0.173 (0.12–0.24) | 0.166 (0.10–0.24) | 0.700 |
| 2 | Lymph | 1.395 (1.01–1.85) | 1.070 (0.90–1.38) | 0.013 |
| 2 | CD8^+^ cell | 0.272 (0.20–0.34) | 0.309 (0.26–0.34) | 0.043 |
| 2 | CD4^+^/PD-1 | 0.053 (0.03–0.11) | 0.039 (0.02–0.08) | 0.136 |
| 3 | Lymph | 1.455 (1.02–1.86) | 1.020 (0.85–1.21) | 0.028 |
| 4 | Lymph | 1.44 (1.16–1.88) | 0.84 (0.79–0.86) | 0.002 |
| 4 | CD8^+^/PD-1 | 0.035 (0.01–0.15) | 0.009 (0.01–0.01) | 0.036 |
| 5 | CD4^+^ cell | 0.370 (0.28–0.41) | 0.260 (0.20–0.27) | 0.030 |
| 5 | CD8^+^ cell | 0.261 (0.22–0.34) | 0.344 (0.30–0.48) | 0.046 |
| 6–9 | — | — | — | — |
